# miR-1273h-5p suppresses CXCL12 expression and inhibits gastric cancer cell invasion and metastasis

**DOI:** 10.1515/med-2022-0486

**Published:** 2022-05-16

**Authors:** Yi-Chen Wang, Song Lu, Xiao-Jiang Zhou, Li Yang, Ping Liu, Lan Zhang, Yuan Hu, Xian-Zhe Dong

**Affiliations:** Department of Pharmacy, Medical Supplier Center, Chinese PLA General Hospital, Beijing 100853, China; Department of Pharmacy, Xuanwu Hospital of Capital Medical University, Beijing 100053, China; College of Pharmacy, Zunyi Medical University, Zunyi 563000, China; Department of Pharmacy, Medical Supplier Center, Chinese PLA General Hospital, No. 28 FuXing Road, Haidian District, Beijing 100853, China; Department of Pharmacy, Xuanwu Hospital of Capital Medical University, 45 Changchun Road, Xicheng District, Beijing 100053, China

**Keywords:** gastric cancer, miR-1273h-5p, proliferation, apoptosis, migration, invasion

## Abstract

The aim of this study was to verify the biological function of miR-1273h-5p in gastric cancer (GC) and its underlying mechanisms. The differential expression of microRNAs between GC and tumor-adjacent normal tissues was detected using microarrays, miR-1273h-5p, and chemokine (C-X-C motif) ligand 12 (CXCL12) mRNA, and protein levels were evaluated using polymerase chain reaction and Western blotting methods, cell proliferation, apoptosis, migration, and invasion were determined by CCK-8, flow cytometry, and transwell assay. Compared to tumor-adjacent normal tissue and gastric epithelial mucosa cell line cells, miR-1273h-5p was significantly downregulated in tissues and cells of GC. The overexpression of miR-1273h-5p could inhibit cell proliferation, migration, invasion, and promote cell apoptosis; in contrast, inhibition of miR-1273h-5p expression could reverse this process. Moreover, a significant upregulation of CXCL12 was observed when the miR-1273h-5p was downregulated in GC cells. Additionally, miR-1273h-5p significantly reduces tumor volume and weight. Thus, this study suggests that miR-1273h-5p regulates cell proliferation, migration, invasion, and apoptosis during GC progression by directly binding to CXCL12 mRNA 3′-untranslational regions, which may be a novel diagnostic and therapeutic target in GC.

## 1 Introduction

The global incidence and mortality rates of gastric cancer (GC) are relatively high [1,2,3], over 70% of cases occur in developing countries [4]. In addition, most GC patients are diagnosed at the advanced stage with extensive involvement of regional lymph nodes or the metastasis to distant organs [5,6] due to non-specific symptoms during the early stage [7]. Moreover, the invasion and metastasis of tumors are the most common reasons for the death from GC [8], and more than 90% of mortality in GC patients is caused by distal metastasis [9]. miRNAs are endogenous non-coding RNAs with a length of about 19–24 nucleotides; numerous studies have confirmed the correlation between the altered expressions of miRNAs and cancer since the dysregulation of miRNAs participates in several important processes during carcinogenesis [10], such as cell growth, differentiation, and apoptosis [11].

Nowadays, many investigations about microRNAs (miRNAs) have been carried out to assess the etiology of GC [12,13,14]. The APEX1/miR-27a-5p axis functions could alter the regulation of the AKT and MAPK pathways serving as a strong potential set of targets for GC-specific chemotherapies [15]. miR-33b-5p also acted as a potential target by downregulating high mobility group AT-hook 2(HMGA2), an indispensable factor that correlated with proliferation and sensitivity to chemotherapy drug of GC cells [16]. miRNA-765 may modify MDR by controlling the expression of BATF2 which provided the possibility of an effective therapeutic target against GC [17] the upregulated miR-106b in cancer-associated fibroblasts of GC patients can accelerate the invasion by regulating phosphatase and tensin homolog deleted on chromosome 10 [18]. Similarly, the roles of miR-106a [19], miR-181b [20], and miR-21 [21] in the development of GC have been identified. Therefore, miRNAs may be a novel therapeutic target for cancers. Nevertheless, the molecular mechanisms underlying the pathogenesis and development of GC remain largely unexplored.

In our current work, we showed that the expression of miR-1273h-5p was dramatically decreased in GC tissues and GC cells; nonetheless, it remained unclear about its function during the progression of GC. So, the present study was to evaluate the antioncogenic function of miR-1273h-5p in GC, which might provide a novel sight for GC therapy.

## 2 Materials and methods

### 2.1 Microarray analysis

A total of 53 pairs of GC and tumor-adjacent normal tissue samples were collected from patients (Table 1) between July 2015 and December 2016, and three pairs of these samples were used for microarray analysis. The tumor-adjacent normal tissues were dissected at 3 cm from the edge of the tumors. Microarray assay and bioinformatics analysis (Gen ontology [GO] and Kyoto Encyclopedia of Genes and Genomes [KEGG] pathway) were performed as described in our previous study [22]. Briefly, approximately 1 µg of total RNA was conjugated with polyA tail using FlashTag™ Biotin HSR Labeling Kit (Affymetrix, Santa Clara, CA, USA). Hybridization was carried out with the labeled RNA at 48°C for 16 h with shaking at 60 rpm for Affymetrix miRNA 4.0 Array. A robust Multichip Analysis algorithm was used to analyze the data. The mRNA–miRNA interaction was predicted using the micode database (http://www.mircode.org/). According to the measured values of mRNA and TarBase database (http://www.microrna.gr/tarbase) forecasts, the miRNA-targeting genes were determined. Combined with the actual differential mRNAs detected using microarray, the negative correlation between miRNA–mRNA was obtained. After predicting the target genes, GO and KEGG pathway analyses were performed. The purpose of these analyses was to identify differentially expressed genes (DEGs) between groups. The data were analyzed using Fisher’s exact test. The *P*-value was calculated using the Benjamini–Hochberg step-up procedure, followed by rectification using the false discovery rate (FDR) method. *P* ≤ 0.05 was regarded as statistically significant. Meanwhile, enrichment scores were adopted to analyze the enrichment level of GO and pathways.

**Table 1:** Clinicopathological features of GC patients

Characteristic		*n* = 53	Proportion (%)
Gender			
	Male	32	60.38
	Female	21	39.62
Age (years)	Average age, 53		
	<60	35	66.04
	≥60	18	33.96
Differentiation			
	Well	36	67.92
	Poor	17	32.08
Tumor size (cm)			
	<5	37	69.81
	≥5	16	30.19
Lymph node metastasis			
	Yes	11	20.75
	No	42	79.25
HP infection			
	Yes	28	52.83
	No	25	47.17
CA125 level (ng/mL)			
	<40	12	22.64
	≥40	41	77.36

HP infection – *H. pylori* infection leading to chronic active gastritis remains the main risk factor for GC. CA125 level – CA125 is most common in the serum of patients with epithelial ovarian tumors. Its positive rate in GC was quite high.

### 2.2 Cell culture conditions

Human GC cell lines that were used in this study (MGC-803, BGC-823, SGC-7901, and MKN-45) and normal gastric epithelial mucosa cell line (GES-1) were provided by Cancer Hospital Chinese Academy of Medical Sciences (Beijing, China). HEK-293 cells were purchased from FuHeng Bio Co., Ltd. (Shanghai, China). GC cells were maintained in RPMI-1640 medium (BioTeke Corporation, Beijing, China), and GES-1 cells were maintained in Dulbecco’s modified Eagle medium (DMEM) in a condition of 37°C containing 5% CO_2_. The medium used for cell culture contained 10% fetal bovine serum (FBS; Thermo Fisher Scientific, Inc., Waltham, MA, USA).

### 2.3 RT-qPCR

GC cells and GES-1 cells were seeded into 6-well plates at a density of 2 × 10^5^ cells/well, after 24 h culture; miRNAs were extracted using miRcute miRNA isolation kit (TIANGEN, Beijing, China). The concentration and purity of miRNA were determined using NanoDrop-2000. The miRNAs were reversely transcribed into complementary DNAs (cDNAs) by miRcute Plus miRNA First-Strand cDNA Synthesis Kit (TIANGEN, Beijing, China), and reverse transcription-polymerase chain reaction (RT-qPCR) was conducted by using miRcute Plus miRNA qPCR Detection Kit (SYBR Green) (TIANGEN, Beijing, China) according to the manufacturer’s instructions. Briefly, initial denaturation step: 94°C for 2 min, melting: 94°C for 20 s, and annealing: 60°C for 34 s. U6 and GAPDH were selected as the housekeeping genes. The primers were synthesized by AuGCT (AuGCT, Beijing, China) as follows: miR-1273h-5p-F: 5′-CTGGGAGGTCAAGGCTGCAGT-3′, miR-1273h-5p-R: 5′-ACTGCAGCCTTGACCTCCCAG-3′; U6-F: 5′-AATCTAGCTGCTGCGGTTCC-3′, U6-R: 5′-GGAACCGCAGCAGCTAGATT-3′; CXCL12-F:5′-CAGATGCCCATGCCGATT-3′, CXCL12-R: 5′TCTGAAGGGCACAGTTTGGA-3′; GAPDH-F: 5′-GTGGAGTCCACTGGCGTCTT-3′, GAPDH-R: 5′-GTGCAGGAGGCATTGCTGAT-3′. The relative expressions of target genes were calculated using the 2^−ΔΔCT^ method.

### 2.4 Western blot

Western blotting analysis was used to analyze the expression of proteins. Briefly, cell lysis was performed using radio immunoprecipitation assay lysis buffer containing protease and phosphatase inhibitor cocktails, and the protein concentrations were determined using a BCA assay. Subsequently, proteins (50 μg) were subjected to sodium dodecyl sulfate-polyacrylamide gel electrophoresis for separation, after which they were electro-transferred onto a polyvinylidene fluoride membrane. After blocking with 5% BSA for 3 h at room temperature, the membranes were incubated with primary antibodies (CXCL12, Proteintech, 17402-1-AP; β-tubulin, Immunoway, YM3030; GAPDH, Immunoway, YM3029) at 4°C overnight followed by incubation with secondary antibodies (HRP-conjugated Affinipure Goat Anti-Rat IgG (H + L), Proteintech, and SA00001-15) for 2 h. Immunoreactive bands were visualized using a chemiluminescence kit, and the density of the bands was determined using scanning densitometry (Bio-Rad, Hercules, CA, USA).

### 2.5 Cell transfection and luciferase reporter assay

As described in our previous studies [23], miR-1273h-5p expression plasmids including mimics, inhibitors, and empty plasmid (GV268) were provided by GeneChem Co., Ltd. (Shanghai, China). In this study, “normal con” cells were not transfected, and “negative con” cells were transfected with an empty vector. The wild-type and mutant CXCL12 plasmids were also prepared by GeneChem Co., Ltd. and cloned into luciferase plasmid GV272 (GeneChem). miRNA mimic is a plasmid that can increase the level of miR-1273h-5p, and miRNA inhibitor can inhibit the endogenous level of miR-1273h-5p. MGC-803, BGC-823, and SGC-7901 cells were cultivated in 96-well plates (5 × 10^3^ cells/well) and then transfected with miR-1273h-5p mimics, inhibitors, or negative plasmids at the final concentration of 1.5 μg/mL. The final concentration of transfection reagent Lipofectamine 2000 (Thermo Fisher Scientific, Inc.) was 0.2 μL/well (96-well plates).

In Luciferase Reporter Assay, HEK-293 cells were seeded into a 96-well plate (1 × 10^4^ cells/well). CXCL12 expression luciferase plasmid (GeneChem Co., Ltd. Shanghai, China) and miR-1273h-5p mimics were co-transfected into HEK-293 cells. Then, pRL-TK Renilla luciferase vectors (Promega, Madison, WI, USA) were transfected. Finally, the luciferase activity was determined using Dual-Glo Luciferase Assay System (Promega). All experiments were repeated three times.

**Figure A1:**
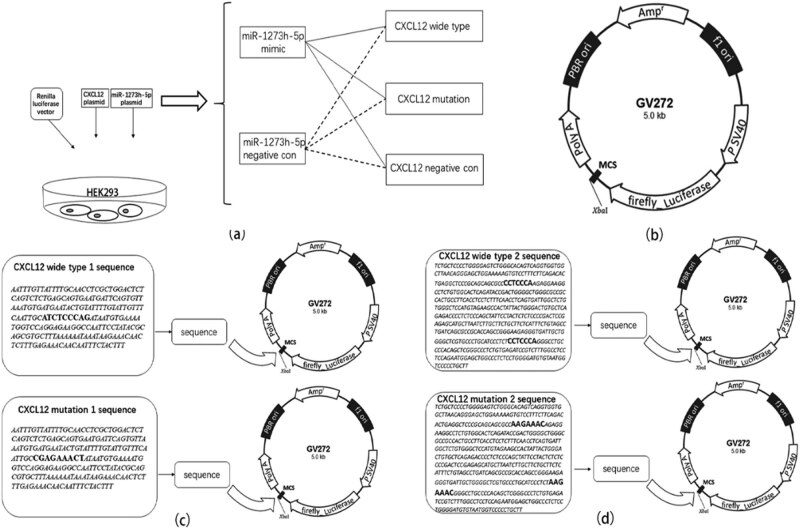
Firefly luciferase plasmids of target gene (CXCL12). (a) During the co-transfection process, experimental grouping has been completed, including miR-1273h-5p(negative con)-CXCL12(negative con), miR-1273h-5p(negative con)-CXCL12(wide type 1 or 2), miR-1273h-5p(negative con)-CXCL12(mutant 1 or 2), miR-1273h-5p(mimic)-CXCL12(negative con), miR-1273h-5p(mimic)-CXCL12(wide type 1 or 2), and miR-1273h-5p(mimic)-CXCL12(mutant 1 or 2). (b) Firefly luciferase negative control plasmids, which did not carry the gene sequence. (c) Base sequences of CXCL12 wild type 1 and mutation 1 and assembly sites with plasmid vectors. (d) Base sequences of CXCL12 wild type 2 and mutation 2 and assembly sites with plasmid vectors. The sequence of junction bases with miRNA is bold.

In the process of constructing target genes, three sequences capable of forming CXCL12 were found distributed in two transcripts. Hence, two plasmids were constructed which were labeled 1,2 at last (Figure A1).

### 2.6 Cell proliferation assay

MGC-803, BGC-823, and SGC-7901 cells were seeded into 96-well plates (5 × 10^3^ cells/well) in 200 μL of RPMI-1640 medium. After 24 h of culture, cells were transfected with 1 μL of miR-1273h-5p mimics or inhibitors (0.3 μg/μL) using Lipofectamine 2000 (Thermo Fisher Scientific, Inc.). Cells in the negative control group were transfected with 1 μL of empty plasmid (0.3 μg/μL). After 24 h incubation, 20 μL of CCK-8 reagent was added to each well according to the manufacturer’s protocols; 4 h later, cell viability was evaluated by using 1420 Multilabel Counter (PerkinElmer, USA) according to the absorbance at 450 nm. Each experiment containing five replicated samples was conducted in triplicate.

### 2.7 Cell apoptosis analysis

GC cells were seeded into 6-well plates (1 × 10^6^ cells/well), followed by 24 h incubation. Subsequently, the cells of “negative con” and “mimics” group cells were transfected with 2 μg/mL of empty plasmid or miR-1273h-5p mimic plasmid, respectively. After 24 h, the cells were collected. After washing with phosphate buffer saline, cells were resuspended with 1X Binding Buffer to achieve a concentration of 5 × 10^6^ cell/mL. Then, cells were stained with Annexin-V FITC and Propidium Iodide Apoptosis Detection Kit (Invitrogen) [24] according to the manufacturer’s protocols. Apoptosis data were analyzed using flow cytometry (FCM; BD Biosciences, Calibur, USA) and Cellquest software. The number of apoptotic cells was equal to the sum of early apoptotic cells and late apoptotic cells. All experiments were conducted in triplicate.

### 2.8 Transwell assays of tumor cell migration and invasion

The transwell assay (Corning, USA) was performed to evaluate cell migratory and invasive capacity of GC cells. For cell invasion assay, 3.35 mg/mL matrigel matrix (CORNING, 354234) was pre-coated on the top of the upper chamber. Cells were suspended at 5.0 × 10^5^ cells/mL in serum-free DMEM, reserving for the next step. Briefly, the upper and bottom transwell chambers (24-well plates) were coated with 100 μL of serum-free medium and 600 μL containing 20% FBS, respectively. The cells were incubated for 24 h. The cells that did not migrate to the lower surface of the membranes were removed from the upper surface of the transwell chamber by a cotton swab. Those migrated cells were stained with 0.1% crystal violet solution. Cell migration assay was the same as invasion assay, except the upper chambers did not need to be coated with Matrigel matrix. Images were then captured under a digital microscope (Olympus IX81, Japan), and the number of cells was counted by the experimenter in five randomly selected fields for each well; every group conducted three wells; all results were presented as mean ± SD.

### 2.9 Xenograft tumor model

Male BALB/c-nu mice (*n* = 30) weighing 14.0–17.0 g, aged 4 weeks, were housed in an environment with a temperature of 22 ± 1°C, relative humidity of 50 ± 1%, and a light/dark cycle of 12/12 h. All animal studies were carried out in compliance with the regulations and guidelines of PLA medical school institutional animal care and conducted according to the AAALAC and the IACUC guidelines.

The mice were randomly divided into three groups after being given adaptive feeding under specific-pathogen-free for 5 days and then subcutaneously injected with 0.2 ml cell suspension (2 × 10^6^ cells/mL in RPMI-1640 containing 10.0% FBS) into the right forearm. The above-mentioned cells fell into three categories: SGC-7901 cells, SGC-7901 cells transfected with miR-1273h-5p mimics, and SGC-7901 cells transfected with miR-1273h-5p-negative plasmid. After 3 weeks, tumor volume and weight were carefully measured. Volume (*V*) was monitored by measuring the tumor length (*L*) and width (*W*) with standard calipers and calculated with the formula of *V* = (*L* × *W* ^2^) × 0.5.

### 2.10 Statistical analysis

SPSS22.0 and GraphPad prism 5.0 (GraphPad Software Inc., San Diego, CA, USA) were adopted for all statistical analyses. Each experiment was repeated three times. Experiment data involving two groups were verified using a *t*-test and expressed as mean ± standard deviation. One-way ANOVA was used for multiple group comparison. Microarray analysis and KEGG pathways of DEGs were analyzed using the standard statistical function of hypergeometric distribution, *t*-test, and FDR. *P* < 0.05 was regarded as statistically significant.


**Ethics approval:** The animal experiment was approved by the Ethics Committee of the Chinese PLA Hospital Medical Laboratory animal Center (China; Approval No. 2019-X15-85). The whole research program including clinical tissue samples has been approved by the Ethics Committee of the Chinese PLA General Hospital.
**Informed consent:** Written informed consent for publication was obtained from all participants.

## 3 Results

### 3.1 Differentially expressed miRNAs in GC tissues and cells

**Figure 1:**
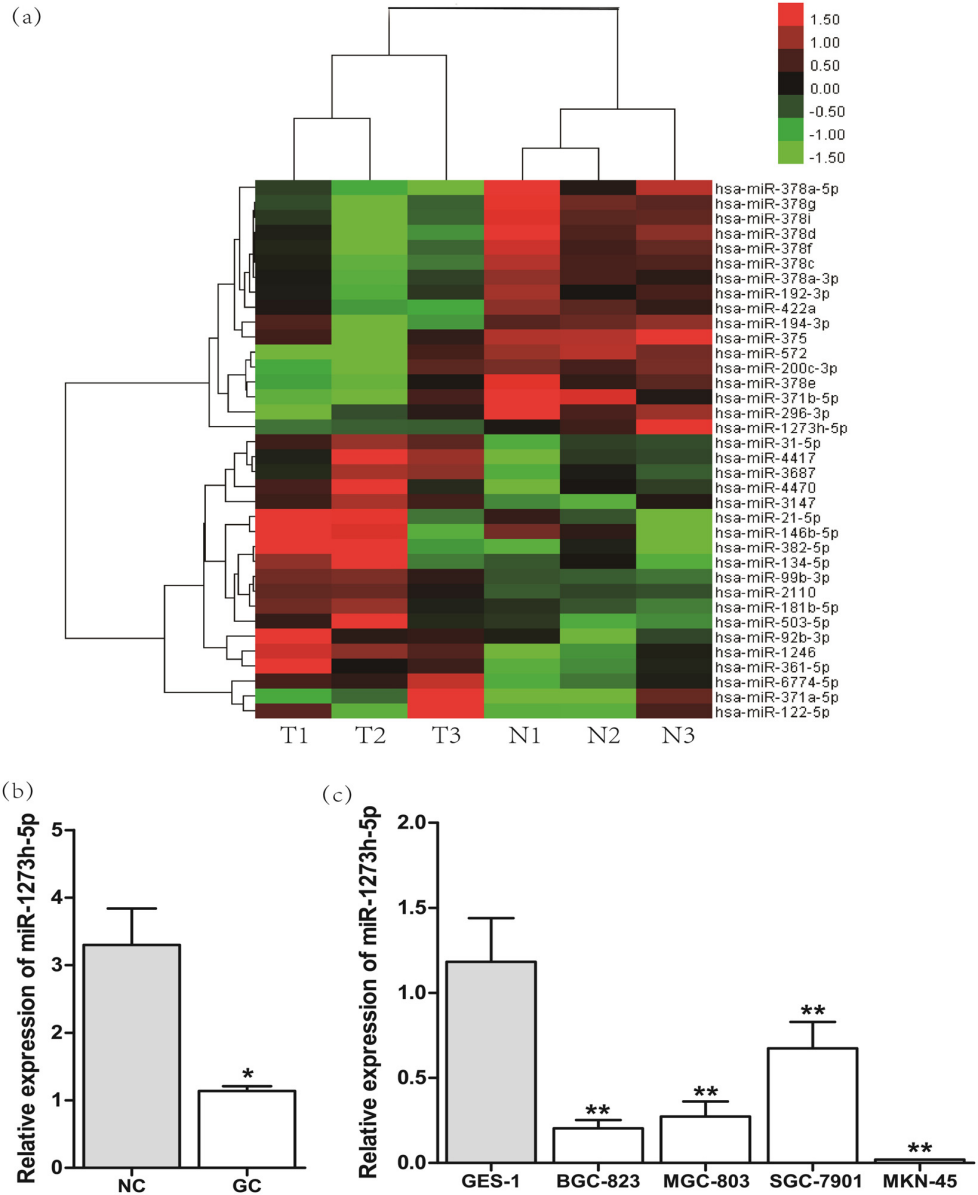
The analysis of differentially expressed miRNAs between GC and tumor-adjacent normal tissues. (a) Cluster analysis of differentially expressed miRNAs between GC and tumor-adjacent normal tissues by microarray assay. Each column indicates a sample, and each row shows the expression level of a gene. The green color indicates low expression, and the red color displays high expression. (b) The relative expression of miR-1273h-5p in 53 pairs of GC and tumor-adjacent NC was analyzed using RT-PCR. (c) The relative expression of miR-1273h-5p in GES-1 and GC cells was determined by RT-PCR. *P < 0.05; compared with GES-1 cells, *P < 0.05, **P < 0.01.

The microarray assay was conducted to identify the differentially expressed miRNAs between gastric tissues and tumor-adjacent normal tissues; 19 miRNAs were overexpressed (ratio, >1.5), and 17 miRNAs were downregulated (ratio, <0.667) in gastric tissues compared with the tumor-adjacent normal tissues (Figure 1a).

Next, the expression of miR-1273h-5p in GC tissues (from 53 GC patients with gastrectomy) was downregulated by 0.31-fold compared with the tumor-adjacent normal tissues (Figure 1b). The expression of miR-1273h-5p in human GC cell lines, such as MGC-803, BGC-823, SGC-7901, and MKN-45 cells, and GES-1 cells was validated using RT-PCR. As shown in Figure 1c, the expression of miR-1273h-5p was decreased in four GC cell lines compared with the GES-1 cell line.

### 3.2 Impact of miR-1273h-5p on the growth and apoptosis of GC cells

**Figure 2:**
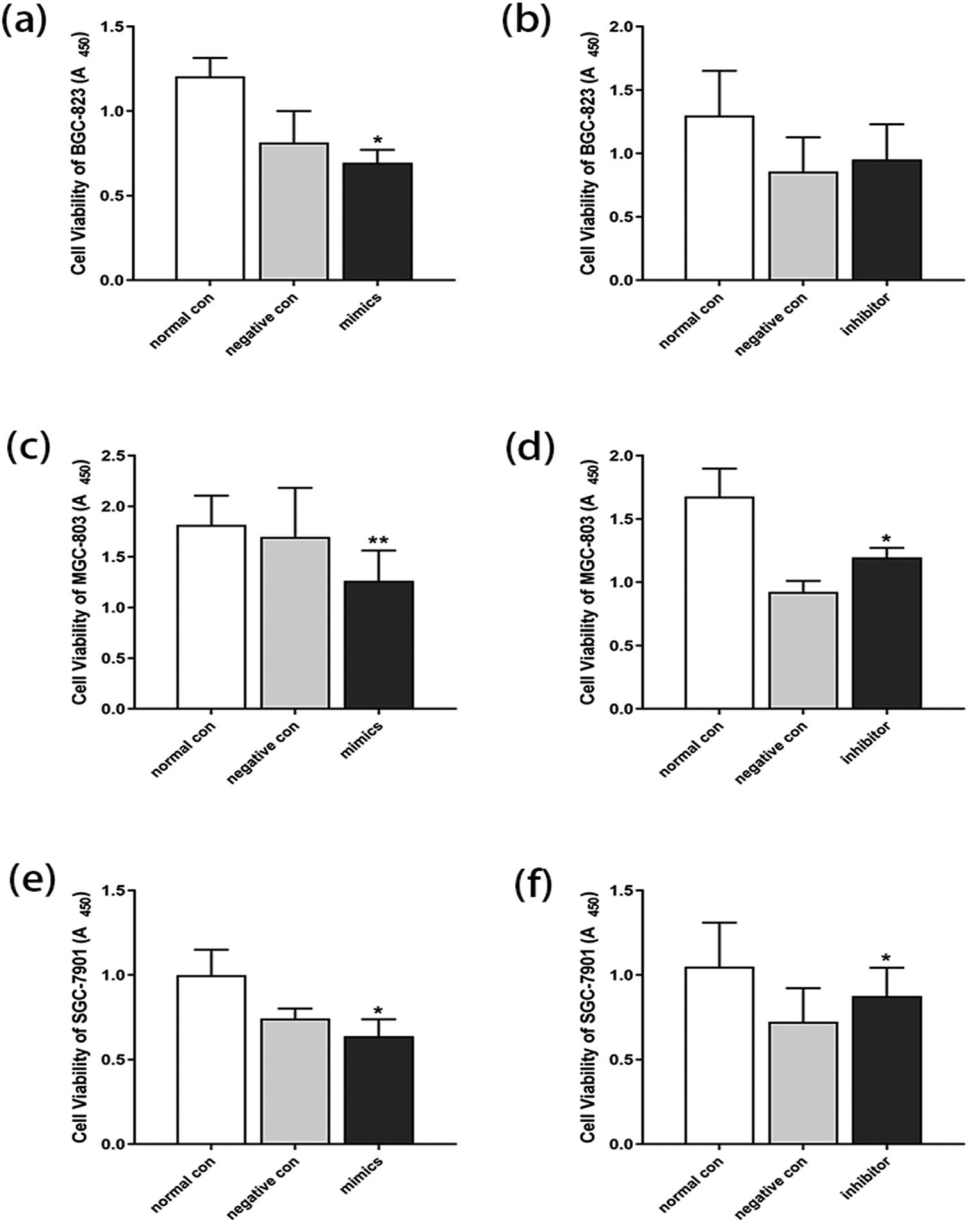
Impact of miR-1273h-5p on GC cell viability. (a and b) Impact of miR-1273h-5p on the proliferation of BGC-823 cells at 24 h after transfection evaluated by CCK-8 assay. Compared to the negative control, the mimic group significantly decreased. (c and d) Effect of miR-1273h-5p on the proliferation of MGC-803 cells at 24 h after transfection evaluated using CCK-8 assay. Compared to negative control group, the proliferation in the mimic group decreased while it increased in the inhibitor group. (e and f) Effect of miR-1273h-5p on the proliferation of SGC-7901 cells at 24 h after transfection evaluated using CCK-8 assay. The mimic group significantly decreased compared to negative control. The inhibitor group had a significant increase in comparison with negative group. All data were expressed as mean ± standard deviation (M ± SD) from three independent experiments compared with the negative control group, *P < 0.05, **P < 0.01.

**Figure A2:**
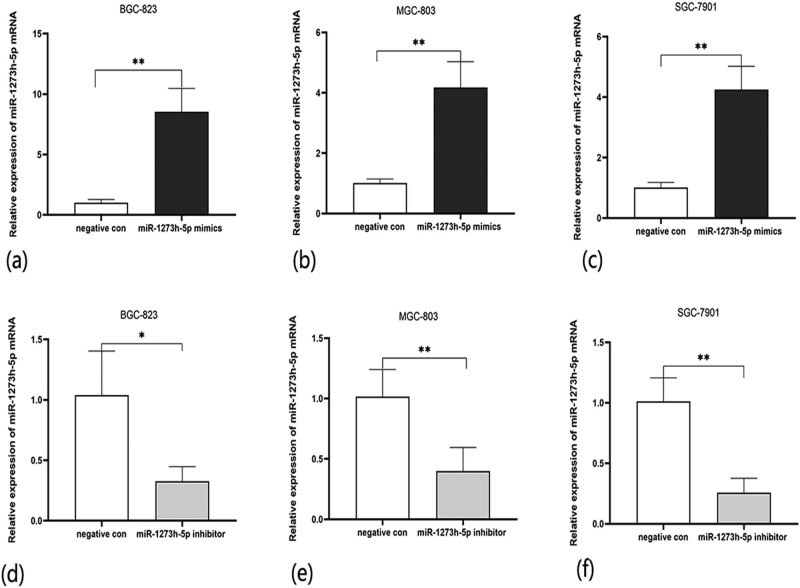
The expression of miR-1273h-5p after miR-1273h-5p mimics and inhibitor transfection. (a–c) The expressions of miR-1273h-5p after transplantation of miR-1273h-5p mimics into BGC-823, MGC-823 and SGC-7901 cells. (d–f) The expressions of miR-1273h-5p after transplantation of miR-1273h-5p inhibitor into BGC-823, MGC-823 and SGC-7901 cells. All data were represented as M ± SD, *P < 0.05,**P < 0.01.

Because the miR-1273h-5p expression was significantly low in GC, we speculated that it might inhibit the growth of GC cells and enhance cell apoptosis. To validate our hypotheses, we transfected miR-1273h-5p mimics in BGC-823, MGC-803, and SGC-7901 cells to upregulate miR-1273h-5p expression and downregulate the miR-1273h-5p expression by transfecting miR-1273h-5p inhibitors into cells. The results of validation confirmed the former hypothesis (Figure A2). Subsequently, a CCK-8 assay was conducted after 48 h of transfection to explore the impact of miR-1273h-5p on the viability of BGC-823, MGC-803, and SGC-7901 cells. When transfected with mimics, the viability of BGC-823, MGC-803, and SGC-7901 cells significantly decreased (Figure 2a, c, and e); in contrast, the viability of cells transfected with miR-1273h-5p inhibitors was increased, especially MGC-803 and SGC-7901 that were significantly increased (Figure 2b, d, and f).

**Figure 3:**
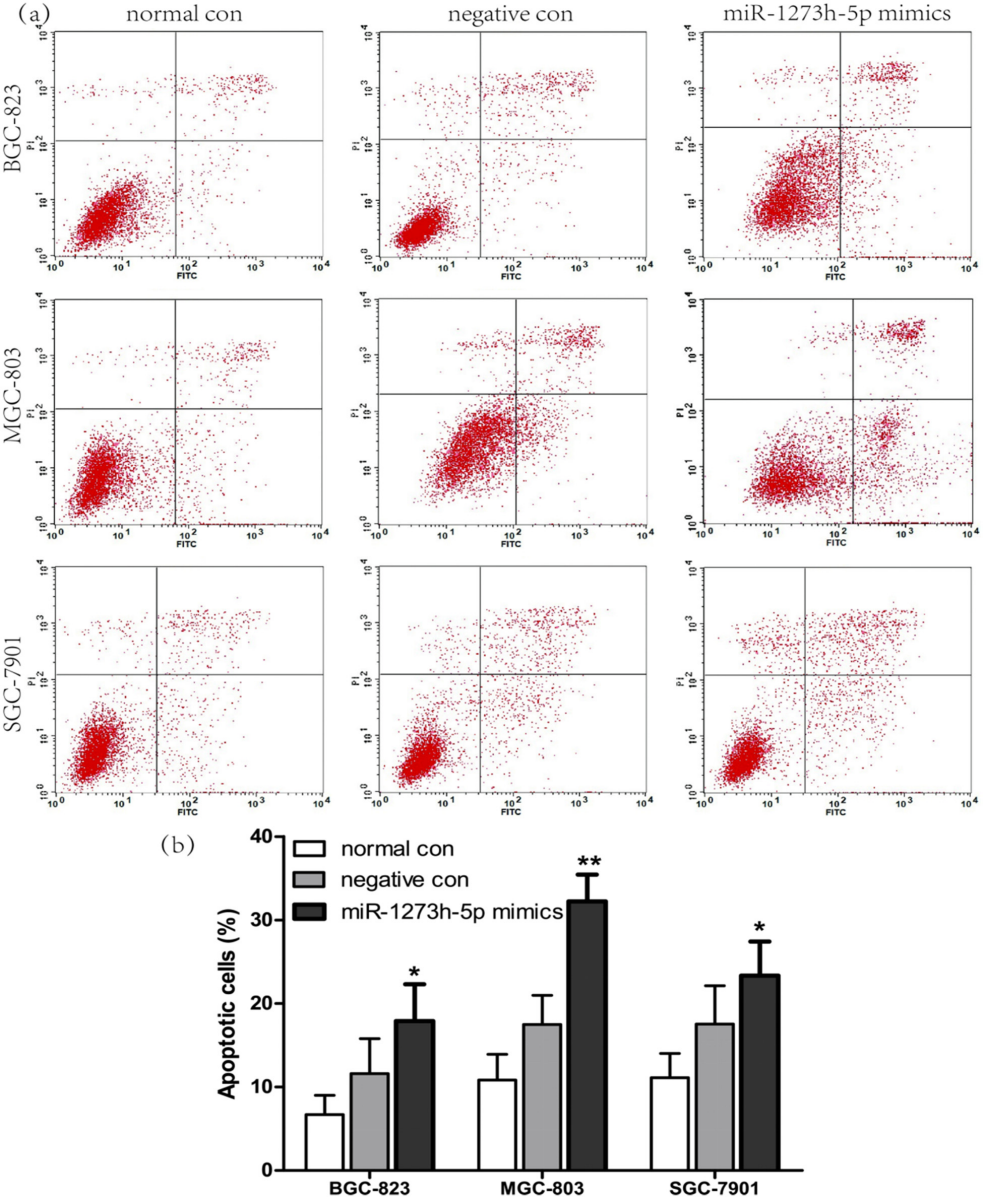
Effect of miR-1273h-5p on GC cell apoptosis. (a) Effect of miR-1273h-5p on apoptosis of BGC-823, MGC-803, and SGC-7901 cells at 24 h after transfection evaluated using FCM. (b) Apoptotic rates of BGC-823, MGC-803, and SGC-7901 cells at 24 h after transfection of miR-1273h-5p mimic. Compared to the negative control, the apoptosis ratio of miR-1273h-5p mimics was significantly increased. All data were expressed as M ± SD from three independent experiments compared with the negative control group, *P < 0.05, **P < 0.01.

PI and Annexin V‑FITC staining were adopted to assess the effect of miR-1273h-5p mimics on cell apoptosis by using FCM (Figure 3a). After the cells were transfected with miR-1273h-5p mimics, the apoptotic rates of BGC-823, MGC-803, and SGC-7901 cells were dramatically enhanced (Figure 3b) compared with the corresponding negative control groups. These findings indicated that miR-1273h-5p had a role in regulating the apoptosis of GC cells.

### 3.3 Effect of miR-1273h-5p on the migration and invasion of GC cells

**Figure 4:**
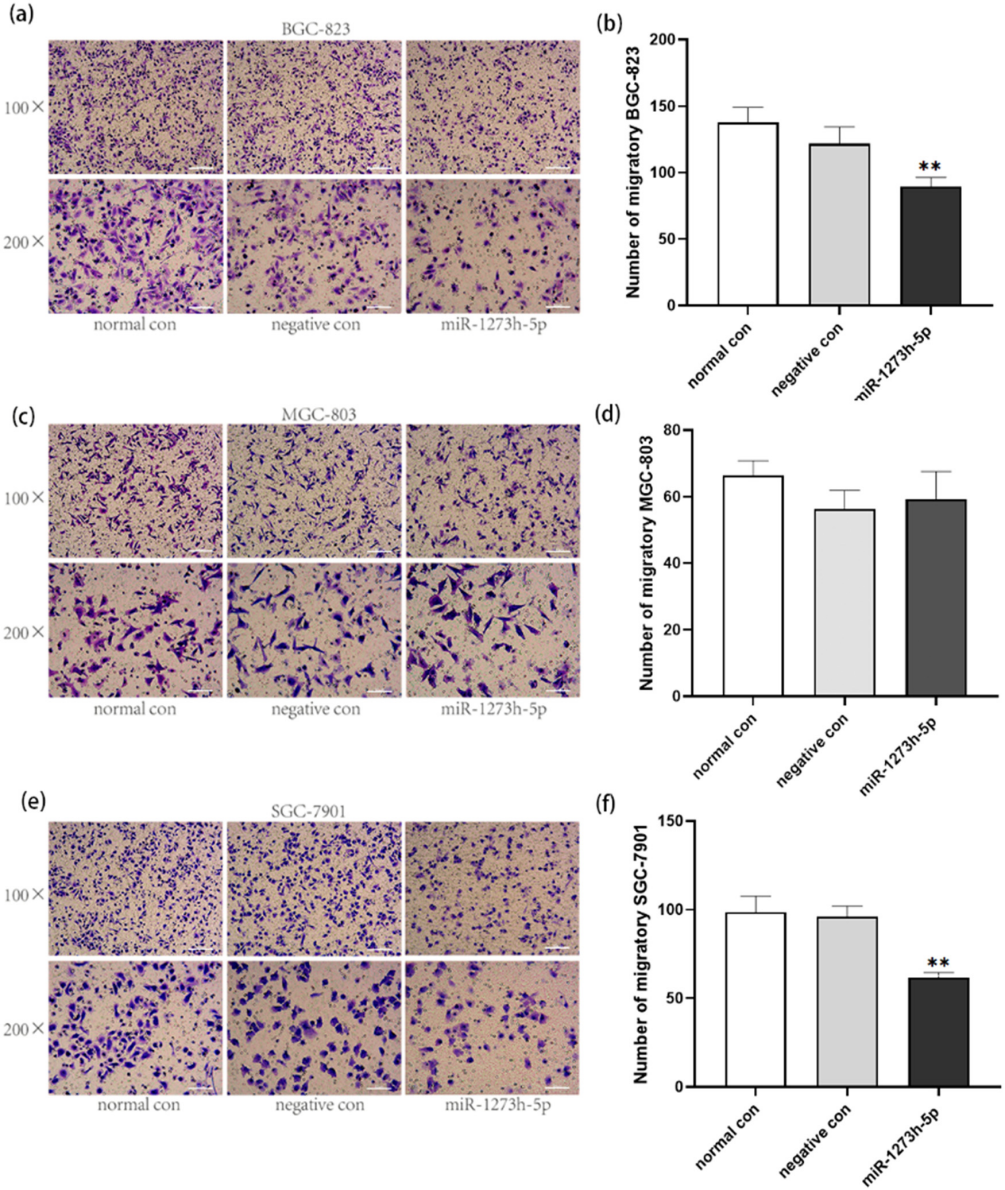
Effect of miR-1273h-5p on GC cell migration. (a and b) Effect of miR-1273h-5p on the migration of BGC-823 cells at 24 h after transfection determined by transwell assay. Compared to the negative control, the ratio of migration was significantly reduced in the miR-1273h-5p group. (c and d) Impact of miR-1273h-5p on the migration of MGC-803 cells at 24 h after transfection determined using transwell assay. (e and f) Impact of miR-1273h-5p on the migration of SGC-7901 cells at 24 h after transfection determined by transwell assay. The migratory ratio of the miR-1273h-5p group revealed significantly decreased contrast with negative control. Pictures of each group included two kinds of specifications with 200× used for statistics. Scale bars have been depicted. All data were expressed as M ± SD from three independent experiments when compared with the negative control group, **P < 0.01.

**Figure 5:**
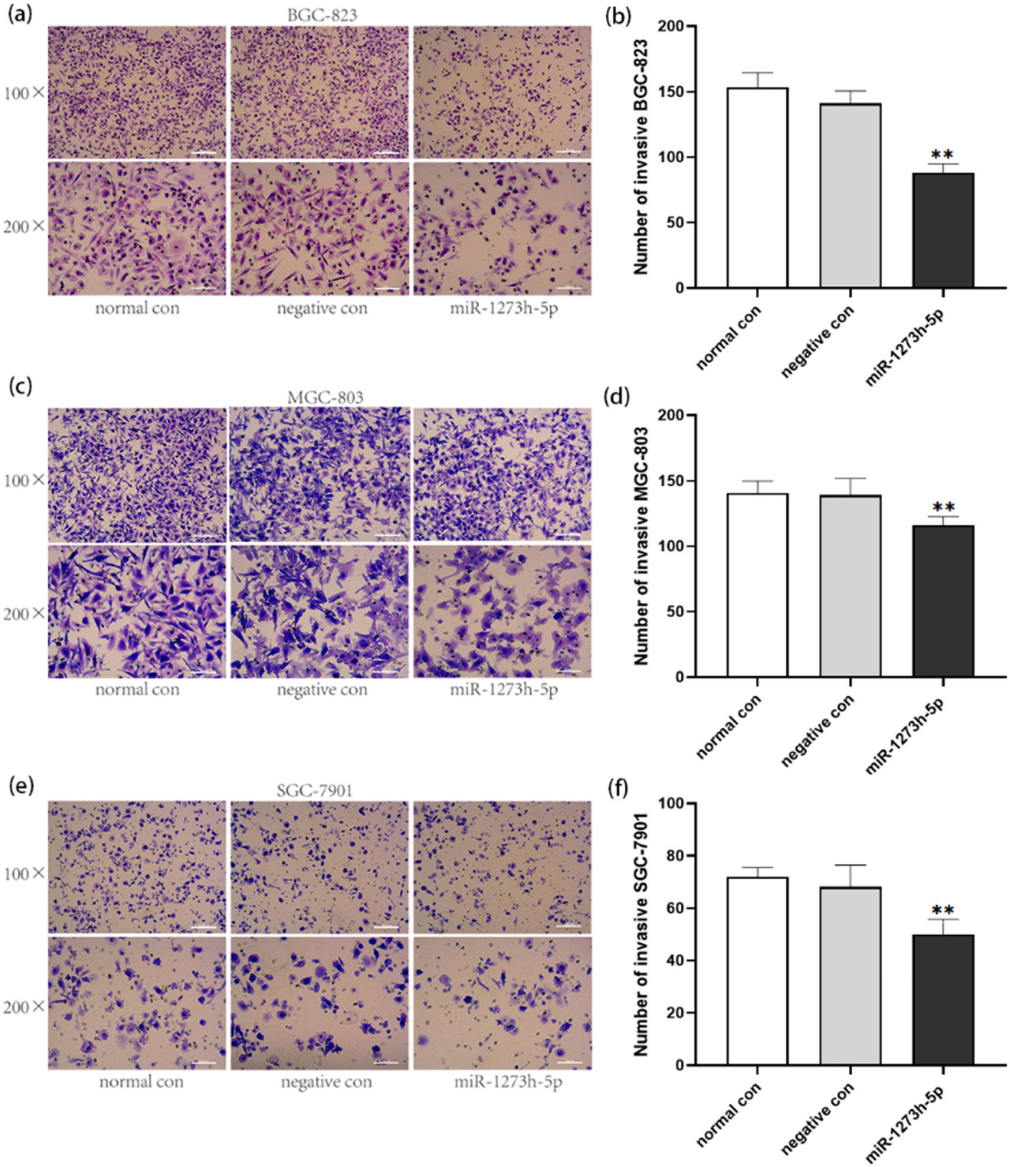
Effect of miR-1273h-5p on GC cell invasion. (a and b) Effect of miR-1273h-5p on the invasion of BGC-823 cells at 24 h after transfection determined by transwell assay. (c and d) Impact of miR-1273h-5p on the invasion of MGC-803 cells at 24 h after transfection determined by transwell assay. (e and f) Impact of miR-1273h-5p on the invasion of SGC-7901 cells at 24 h after transfection determined using transwell assay. The miR-1273h-5p group had a significant decrease in contrast with the negative group in three cell models. Pictures of each group included two kinds of specifications with 200× used for statistics. Scale bars have been depicted. All data were expressed as M ± SD from three independent experiments compared with the negative control group, **P < 0.01.

Cell migration and invasion were explored using the transwell assay, and the number of migratory and invasive cells was counted using a microscope (100× and 200×). Results indicated that the overexpression of miR-1273h-5p inhibited the migration of BGC-823 and SGC-7901 cells (Figure 4). In addition, the invasion of BGC-823, MGC-803, and SGC-7901 cells was also inhibited by overexpression of miR-1273h-5p compared to normal and negative control groups (Figure 5).

### 3.4 High expression of miR-1273h-5p downregulates CXCL12

**Figure 6:**
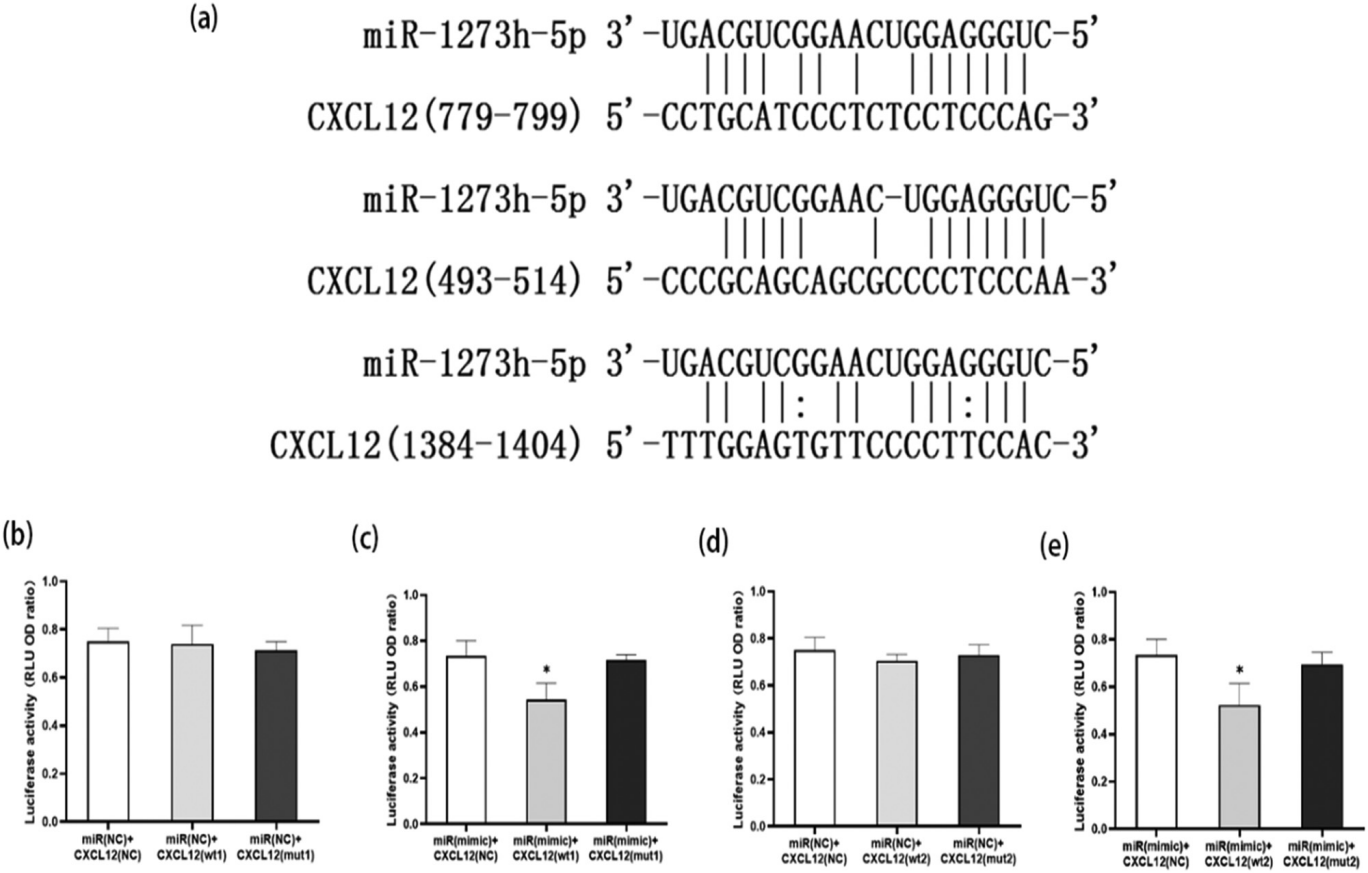
Bioinformatics analysis of miR-1273h-5p and target gene correlation. (a) Potential targeting (binding) site of miR-1273h-5p on CXCL12 mRNA predicted using TargetScan and miRanda software. (b and d) After the combination of miR-1273h-5p-negative control plasmid with CXCL12 plasmids, including negative control, wide type, and mutation. (c and e) In comparison with negative control group, the RLU ratio of miR-1273h-5p mimic combined with CXCL12 wide-type plasmid significantly decreased. All transfection trials were conducted using the HKE293 cells (*P < 0.05).

Bioinformatics analysis was conducted using TargetScan and miRanda software, and CXCL12 was identified as a potential target gene (Figure 6a). And miR-1273h-5p negative control plasmid could not regulate the CXCL12 level (Figure 6b and d). However, overexpression of miR-1273h-5p could downregulate wild-type CXCL12 mRNA level, but could not downregulate mutant-type CXCL12 mRNA (in which binding site was mutated) level (Figure 6c and e).

### 3.5 Binding to the target gene CXCL12 in GC cells

**Figure 7:**
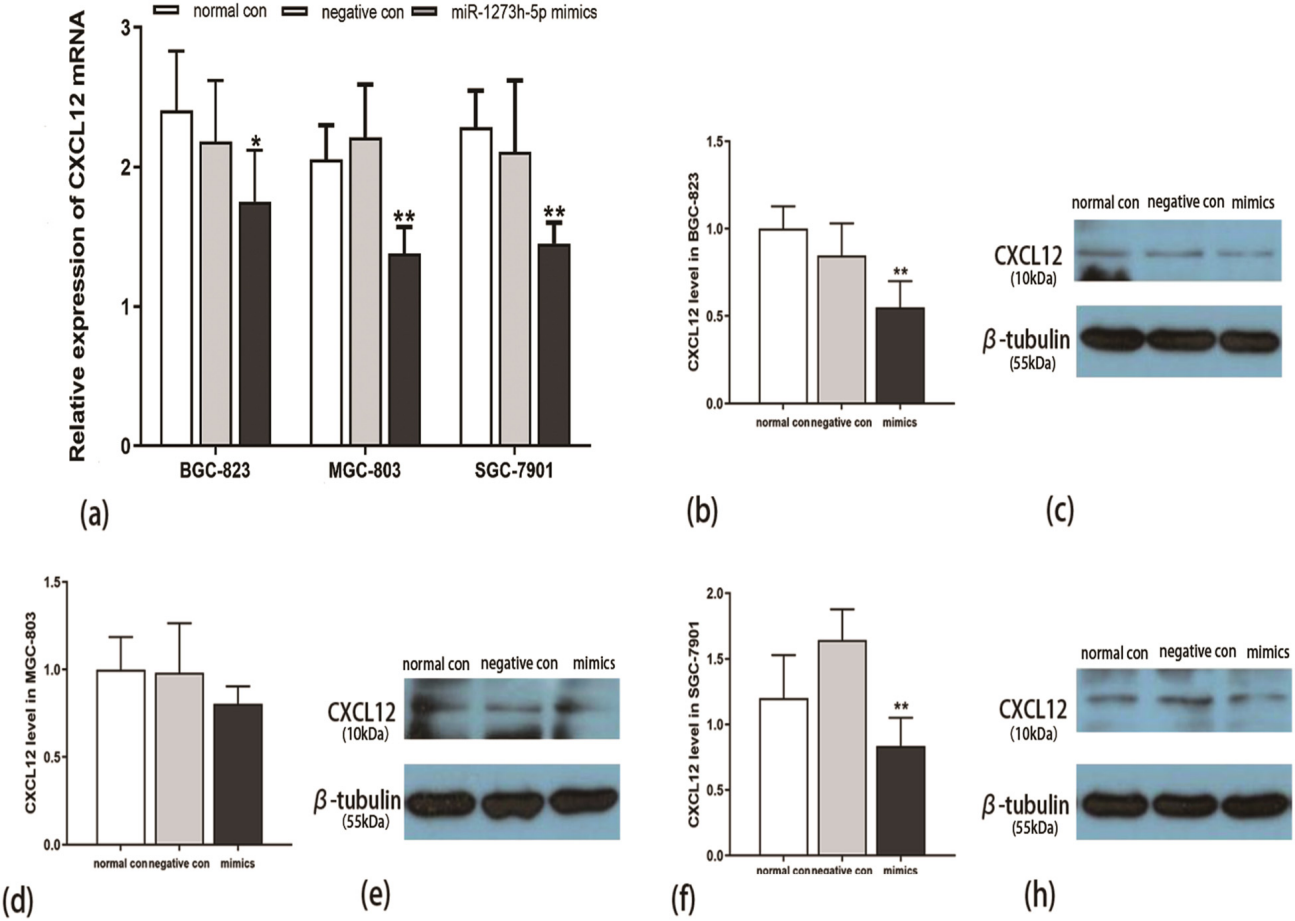
Verification of target gene CXCL12 in GC cells. (a) The expression of CXCL12 at the mRNA level in BGC-823, MGC-803, and SGC-7901 cells following miR-1273h-5p mimic transfection was evaluated using RT-PCR. miR-1273h-5p mimic group revealed a significant decrease compared to the negative group in three GC cells. (b, d, and f) The expression of CXCL12 at the protein level in BGC-823, MGC-803, and SGC-7901 cells following miR-1273h-5p mimic transfection determined using Western blot. The expression of CXCL12 in mimics revealed a significant reduction of BGC-823 and SGC-7901cells by contrast with negative control. (c, e, and h) Protein electrophoretic bands of BGC-823, MGC-803, and SGC-7901 cells, respectively. All data were represented as M ± SD from three independent experiments compared with the negative control group, *P < 0.05, **P < 0.01.

The CXCL12 mRNA expression was dramatically downregulated in GC cells transfected with miR-1273h-5p mimics (Figure 7a). Similarly, the protein expression of CXCL12 was also obviously reduced (Figure 7b–h). These results suggested that miR-1273h-5p may play a biological role in down-regulating the expression of CXCL12.

### 3.6 *In vivo* validation

**Figure 8:**
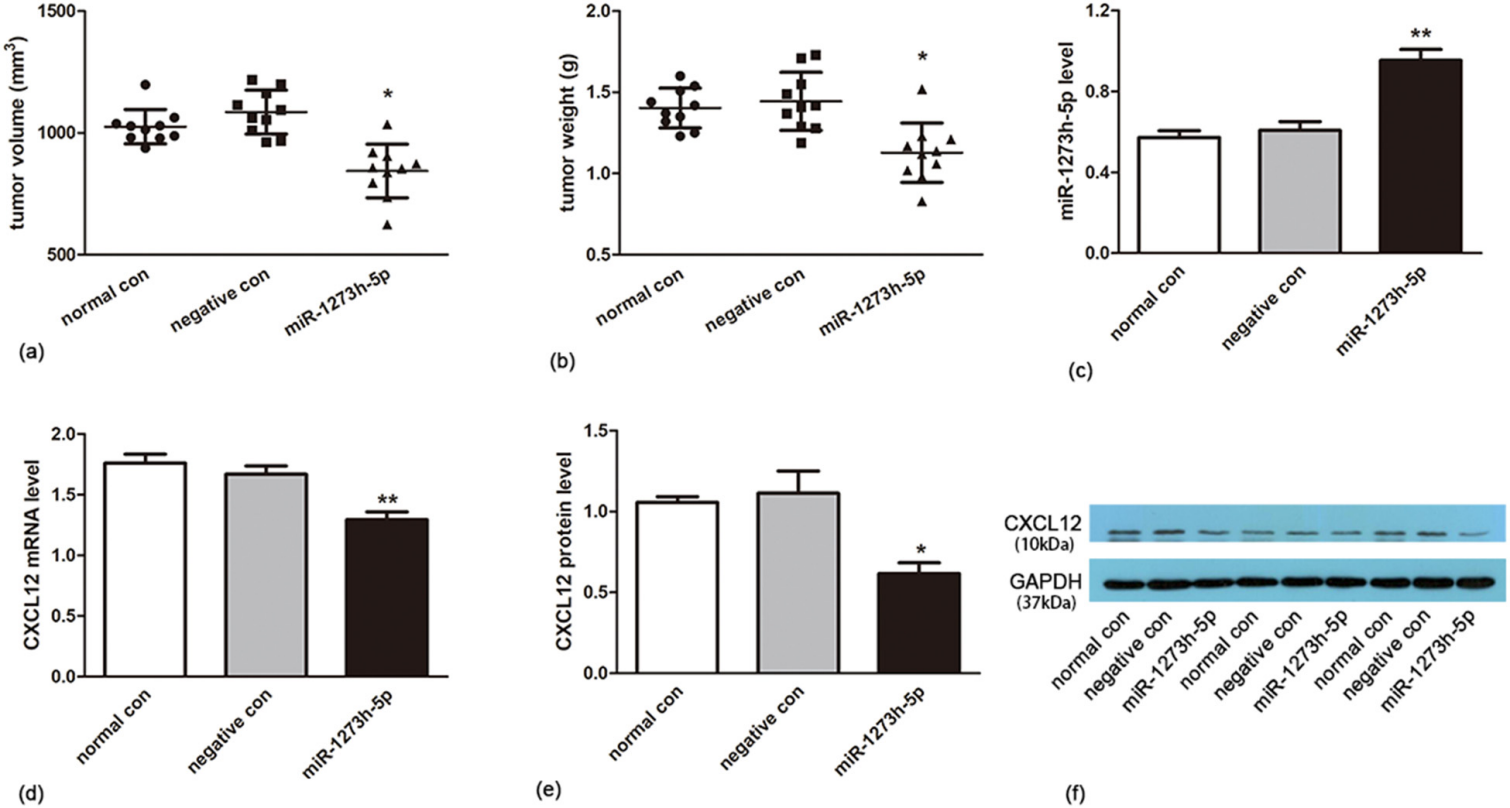
Effect of miR-1273h-5p on SGC-7901 cell transplanted mice model. (a and b) Tumor volume and weight of cancer tissue among the normal control group, negative control group, and miR-1273h-5p group revealed significant reductions. (c and d) The expression of miR-1273h-5p and CXCL12 at the mRNA level in normal control, negative control, and the miR-1273h-5p transfected groups was evaluated using RT-PCR. In contrast with the negative group, the miR-1273h-5p group significantly increased. (e and f) The expression of CXCL12 at the protein level in three groups was determined by Western blotting analysis. The miR-1273h-5p group significantly decreased compared to the negative group. All data were represented as M ± SD from three independent experiments compared with the negative control group, *P < 0.05, **P < 0.01.

The xenograft tumor model in nude mice was established, and the volume and weight of tumor tissue were measured after 21 days of feeding. The tumor volume and weight of the miR-1273h-5p mimic group (*n* = 10) were significantly lower than those of the normal control group (*n* = 10) and negative control group (*n* = 10) (Figure 8a and b), and the miR-1273h-5p level was significantly overexpressed (Figure 8c). CXCL12 mRNA and protein were significantly lower in the miR-1273h-5p group compared to normal and negative control groups (Figure 8d–f).

## 4 Discussion

It is well known that the dysfunction of miRNAs is tightly correlated with the pathogenesis of different human tumors, thus indicating that miRNAs may function as oncogenes or tumor suppressors [25]. For example, the overexpression of miR-215 leads to the suppressed growth and penetration of breast cancer cells by targeting AKT1 [26]. The upregulated miR-223-3p enhances the cell proliferation, migration, and invasion in ovarian cancer by targeting SOX11 [27]. miRNA-3662 is a negative regulator of pancreatic ductal adenocarcinoma (PDAC) chemoresistance by constructing the PDAC subcutaneous xenograft tumor model [28]. Co-delivery of 5-FU and miR-34a(m) could achieve synergistic effects on tumor suppression *in vitro* and *in vivo* by enhancing anti-colorectal cancer (CRC) activity of 5-FU via silencing sirt1 expression and suppressing CRC cell migration by targeting CD44 [29].

In GC, many dysregulated miRNAs, such as miR-30b, miR-372, and miR-21, have been shown to be involved in the growth, apoptosis, migration, and penetration [30,31,32]. In our current work, miR-1273h-5p was lowly expressed in GC tissues and cell lines, reducing the level of miR-1273h-5p can promote the proliferation of GC cells, suggesting that miR-1273h-5p may play a tumor suppressor role in GC. If this hypothesis is correct, increasing the level of miR-1273h-5p may play a role in suppressing GC. After we exogenously increased intracellular miR-1273h-5p, the proliferation, invasion, and migration ability of GC cells, and the growth of transplanted tumors were significantly decreased, meanwhile the apoptosis of cells was increased, indicating the tumor suppressor function of miR-1273h-5p. These findings provide new clues for GC treatment.

miR-1273h-5p is a member of the miR-1273 family, and the expression of miR-1273 is frequently found in various kinds of diseases. miR-1273d and miR-1273g-3p have been associated with different types of cancers, such as progressive lymphoma, diffused melanoma neoplasm metastasis, neoplasm skin neoplasms, uterine, and cervical neoplasms [33]. Moreover, miR-1273g-3p is also significantly dysregulated in patients with chronic obstructive pulmonary disease [34]. The upregulation of miR-1273 was detected in the KrasG12D Pdx1-Cre pancreatic cancer mouse model compared with the control mice [35]. The downregulation of miR-1273 in early atherosclerotic plaque tissues has been confirmed, and the specific regulatory pattern of miRNAs in early atherosclerotic plaques may be useful in determining the formation and stability of plaques [36].

MiRNAs can modulate the translation and stability of their target messenger RNAs (mRNAs) by binding to complementary sequences of 3′-untranslational regions (3′-UTR) of the transcripts [37]. miRNA duplex was generated through a sophisticated biochemical process from the pre-miRNA. After integrating with RNA-induced silencing (RISC) complex, the duplex guided the RISC complex to the target mRNA aiming at a complementarily dependent process-regulating genes. The results of complement between “seed region” and target mRNA could lead to two consequences: related RISC’s degradation and translation repression [38].

By analyzing the binding sites and co-expression, we found that Chemokine 12 (CXCL12) has binding sites with miR-1273h-5p, and the expression is negatively correlated, which may be the target gene of miR-1273h-5p. Previous studies have found that CXCL12 can be regulated by miRNAs. miR-448 inhibits cell proliferation, migration, and invasion in ovarian cancer by targeting CXCL12 expression [39]. Some complementary binding sites between miR-455-5p and CXCL12 have been found, and a significant inverse correlation has been detected as well, indicating that miR-455-5p might suppress medullary thyroid cancer growth and metastasis by targeting CXCL12/CXCR4 signaling pathway [40]. In GC, miR-204-5p is discovered to target the 3′-UTR of CXCL12 as a tumor suppressor, regulating invasion and migration [41]. miR-23a-3p could modulate CXCL12-mediated angiogenesis to affect GC’s proliferation and migration [42]. The increasing expression of CXCL12 under the interaction of miR-141-3p and circDLG1 could promote GC progression and resistance to anti-PD-1-based therapy [43].

CXCL12 is also known as stromal cell-derived factor-1, a member of the CXC chemokine subfamily. It maintains tissue homeostasis in different physiological and pathological processes and participates in the survival and recruitment of immune cells [44,45,46]. Numerous investigations have shown that extracellular CXCL12 is overexpressed in different types of tumors and promotes the occurrence, invasion, and metastasis of tumors such as GC, CRC, breast cancer, and melanoma [47]. The combination of CXCL12 and CXCR4 can activate signaling pathways such as MAPK/ERK, PI3K/Akt/NF-κB, and c-Jun N-terminal kinase and regulate tumor progression [48,49]. Overexpression of CXCL12 can promote the growth of human breast cancer cells [50], exacerbating nasopharyngeal carcinoma cell migration and invasion by binding to its receptor CXCR4 [51].

In GC tissues, CXCL12/CXCR4, highly overexpressed, is tightly correlated with the metastasis of lymph nodes, higher tumor, node, metastasis staging, and poor prognosis [52,53]. CXCL12 can also promote the expressions of epidermal growth factor receptor ligands, such as amphiregulin and heparin-binding EGF-like growth factor, in GC cells leading to increased migration [54]. CXCL12 could also mediate the trafficking of normal and tumor cells by binding to CXCR7 [55], and the CXCL12/CXCR7 axis is involved in lymph node and liver metastasis of GC [56].

Collectively, miR-1273h-5p functions as a tumor suppressor gene and participates in the pathogenesis of GC. Low-expressed miR-1273h-5p in GC may relieve its inhibitory effect on CXCL12. In another aspect, overexpression of miR-1273h-5p could enhance the apoptosis of GC cells and suppress the cell growth and invasion, possibly by binding to 3′-UTR of CXCL12 mRNA so as to decrease the CXCL12 expression. Therefore, miR-1273h-5p may be a new therapeutic regimen for GC patients in the future.
